# Supplementary material for: A simplified method for blood feeding, oral infection, and saliva collection of the dengue vector mosquitoes
Source: PLoS One. 2020 May 29;15(5):e0233618. doi: 10.1371/journal.pone.0233618 (PMC7259494; doi:10.1371/journal.pone.0233618)
Supplement: S1 Table — (DOCX) [file pone.0233618.s001.docx]

**Table S1.** Blood-feeding efficiency of artificial blood feeder compared with mice blood-fed female mosquitoes

| **Replicate** | **Total engorged mosquitoes** | | | |
| --- | --- | --- | --- | --- |
|  | ***Ae. aegypti*** | | ***Ae. albopictus*** | |
|  | **Mice** | **Artificial feeder** | **Mice** | **Artificial feeder** |
| Exp.1 | 60% (30/50) | 80% (40/50) | 84% (42/50) | 88% (44/50) |
| Exp.2 | 56% (28/50) | 90% (45/50) | 88% (44/50) | 86% (43/50) |
| Exp.3 | 64% (32/50) | 84% (42/50) | 90% (45/50) | 82% (41/50) |
| Average of percentages | 60% | 84.67% | 87.33% | 85.33% |

Note: Each replicate shows an example with n=50.
