# Supplementary material for: A simplified method for blood feeding, oral infection, and saliva collection of the dengue vector mosquitoes
Source: PLoS One. 2020 May 29;15(5):e0233618. doi: 10.1371/journal.pone.0233618 (PMC7259494; doi:10.1371/journal.pone.0233618)
Supplement: S2 Table — (DOCX) [file pone.0233618.s002.docx]

**Table S2.** Eggs laid efficiency of individual female mosquito that fed on artificial blood feeder or mice blood-fed

| **Replicate** | ***Ae. aegypti*** | | ***Ae. albopictus*** | |
| --- | --- | --- | --- | --- |
|  | **Mice** | **Artificial feeder** | **Mice** | **Artificial feeder** |
| Exp.1 | 108.2 ± 3.65 | 104.4 ± 3.41 | 109.9 ± 3.99 | 108.9 ± 3.94 |
| Exp.2 | 111.9 ± 3.99 | 105.95 ± 3.66 | 108.65 ± 3.54 | 103.4 ± 3.41 |
| Exp.3 | 105.65 ± 3.54 | 106.9 ± 3.94 | 112.2 ± 3.65 | 106.95 ± 3.66 |
| Average of  eggs laid/mosquito | 108.58 ± 1.81 | 105.75 ± 0.72 | 110.25 ± 1.03 | 106.42 ± 1.02 |

Note: Each replication represented the eggs laid of 20 mosquitoes and presented as mean ± SEM.
