# Supplementary material for: A simplified method for blood feeding, oral infection, and saliva collection of the dengue vector mosquitoes
Source: PLoS One. 2020 May 29;15(5):e0233618. doi: 10.1371/journal.pone.0233618 (PMC7259494; doi:10.1371/journal.pone.0233618)
Supplement: S3 Table — (DOCX) [file pone.0233618.s003.docx]

**Table S3.** Comparison of saliva protein concentration that collected from different number of mosquitoes using artificial feeder

| **Total mosquitoes** | **Saliva protein concentration (µg/ml)** | |
| --- | --- | --- |
|  | ***Ae. aegypti*** | ***Ae. albopictus*** |
| 1 | - | - |
| 10 | - | - |
| 20 | 21.57 ± 1.94 ^a^ | 16.3 ±2.44 ^a^ |
| 50 | 87.95±1.51 ^b^ | 67.71 ± 4.82 ^b^ |
| 100 | 185.35±1.68 ^b^ | 175.43 ±3.06 ^b^ |
| 150 | 289.29±7.79 ^b^ | 257.78 ± 1.04 ^b^ |

Note: Data were pooled from three independent experiments and presented as mean ± SEM. a, base values for comparison with other values; ab, not significant; b, P < 0.005 (Unpaired *t*-test).
