# Supplementary material for: A simplified method for blood feeding, oral infection, and saliva collection of the dengue vector mosquitoes
Source: PLoS One. 2020 May 29;15(5):e0233618. doi: 10.1371/journal.pone.0233618 (PMC7259494; doi:10.1371/journal.pone.0233618)
Supplement: S4 Table — (DOCX) [file pone.0233618.s004.docx]

**Table S4.** Comparison of saliva titers collected from DENV2-infected *Ae. aegypti* via artificial feeder at different days post infection

| **Days post infection** | **Average number of engorged mosquitoes** | **Average**  **feeding rate (%)** | **Log10 plaque forming units (PFU/ml)** |
| --- | --- | --- | --- |
| 3 | 162 ± 1.15 ^a^ | 81 ± 0.58 ^a^ | - |
| 5 | 168 ± 1.15 ^ab^ | 84 ± 0.58 ^ab^ | - |
| 7 | 164.67 ± 1.76 ^ab^ | 82.33 ± 0.88^ab^ | 1.13±0.56 ^a^ |
| 10 | 165 ± 1.52 ^ab^ | 82.5 ± 0.76 ^ab^ | 2.45±0.36 ^ab^ |
| 14 | 162.67 ± 0.95 ^ab^ | 81.33 ± 0.47^ab^ | 3.44±0.25 ^b^ |

Note: Data were pooled from three independent experiments and presented as mean ± SEM. Total 200 mosquitoes were used for each replicate. a, base values for comparison with other values; ab, not significant; b, *P* < 0.05 (Unpaired *t*-test).
