# Supplementary material for: A simplified method for blood feeding, oral infection, and saliva collection of the dengue vector mosquitoes
Source: PLoS One. 2020 May 29;15(5):e0233618. doi: 10.1371/journal.pone.0233618 (PMC7259494; doi:10.1371/journal.pone.0233618)
Supplement: Attachment — Submitted filename: Review Comments - Simplified Method for Blood Feeding.docx [file pone.0233618.s005.docx]

Lines 35-37 need some revising; the current sentence structure is complicated and confusing.

Lines 40-43 needs to be revised; again, the current sentence structure is confusing and hard to follow.

Line 55 consider changing to read as; …feeding these vectors due to ethical approval, …

Line 57 might read better as; …using live animals is costly and maintaining them is laborious.

Line 58 should read; Artificial blood-feeding systems are important…

Line 60 consider revising as; …feed blood-sucking insects [6-7], most of them …

Lines 61-62 consider revising as; …thin membranes filled with warmed animal blood, that allow female mosquitoes to insert their proboscis to acquire a blood meal.

Lines 67-74 these sentences need revising

Line 83 consider revising as; Mouse-use for blood …

Line 85 consider revising as; BALB/c mice were obtained …

Line 86 should read; …Co., Ltd., all mice were …

Line 90 should read; …dose of 75mg/kg i.p. during the blood feeding experiment …

Line 100 might read better as; **Mosquito blood-feeding and egg laying experiments**

Line 111 the authors state that they determined if mosquitoes had taken a full meal and then calculated the percentage of engorged females. Could the authors further elaborate on what is meant by a full meal and the criteria used to determine this?

Line 125 should read as; …washed sheep erythrocytes, …

Lines 126-127 this sentence is hard to follow, please consider rephrasing

Lines 150-153 are hard to follow, please revise this section

Line 153 consider changing to; The sterilized saliva-containing …

Line 158 unsure what is meant by this sentence, could the authors either give a brief explanation of what is meant or revise this sentence?

Line 174 change to; … artificial blood feeder compared to live animals, …

Line 176 change to; …compare the efficiency between the two different blood …

Line 178 change to; …compared to 60% of females fed on …

Lines 183-184 Could the authors explain what they are trying to state with this sentence? I am unsure if they mean that albopictus have a preference for human blood or if their preference is for other mammals?

Line 190 should read; …for female mosquito blood feeding.

Lines 203-204 This sentence needs rephrasing

Line 208 should read; …the number of engorged *Ae. aegypti*…

Line 215 should read as; To infect with virus, *Ae. aegypti* consumed infectious blood using …

Lines 216-218 these sentences are hard to follow, please consider rephrasing

Line 229 should read; … we could collect sufficiently infected saliva…

Lines 244-246 Could the authors expound what is meant here, it is unclear to me? Are they describing limitations of other experiments are based on the sample size of mosquitoes used? Or implying that number of mosquitoes in a single container is the limiting factor?

Lines 321-322 this is confusing, please revise this sentence

Line 345 should read; …saliva was collected at 3, 5, 7, 10, and 14 days post infection via…

Lines 347-348 should read; …DENV-2 infected mosquito saliva at different days post infection.

Line 349 should read; …mosquito saliva titer at different days post infection…
